# Differential regulation of neurexin at glutamatergic and GABAergic synapses

**DOI:** 10.3389/fncel.2013.00035

**Published:** 2013-04-04

**Authors:** Giulia Pregno, Elena Frola, Stefania Graziano, Annarita Patrizi, Federico Bussolino, Marco Arese, Marco Sassoè-Pognetto

**Affiliations:** ^1^Department of Neuroscience, University of TurinTorino, Italy; ^2^Department of Oncological Sciences and Institute for Cancer Research and Treatment, University of TurinCandiolo, Italy; ^3^F.M. Kirby Neurobiology Center, Harvard Medical School, Children's HospitalBoston, MA, USA

**Keywords:** neurexin, neuroligin, GABA_A_ receptor, synaptic specificity, cerebellum

## Abstract

Neurexins (Nrxs) have emerged as potential determinants of synaptic specificity, but little is known about their localization at central synapses. Here we show that Nrxs have a remarkably selective localization at distinct types of glutamatergic synapses and we reveal an unexpected ontogenetic regulation of Nrx expression at GABAergic synapses. Our data indicate that synapses are specified by molecular interactions that involve both Nrx-dependent and Nrx-independent mechanisms. We propose that differences in the spatio-temporal profile of Nrx expression may contribute to specify the molecular identity of synapses.

## Introduction

The assembly, validation, and specificity of synapses are thought to depend on *trans*-synaptic interactions between cell-adhesion molecules (Waites et al., 2005; Shen and Scheiffele, 2010; Williams et al., 2010). Neurexins (Nrxs) are among the best characterized adhesion molecules that have been implicated in synapse formation and synaptic specificity (Ichtchenko et al., 1995; Craig and Kang, 2007). When expressed in non-neuronal cells, Nrxs induce synapse formation on co-cultured neurons, suggesting that they function in the initial assembly of synaptic connections (Graf et al., 2004; Nam and Chen, 2005). Deletion of α-Nrxs in mice results in a lethal phenotype characterized by a massive impairment in Ca^2+^-channel function and neurotransmitter release (Missler et al., 2003; Zhang et al., 2005). In *Drosophila*, mutation of the single Nrx gene causes severe structural defects of the neuromuscular junction, and corresponding alterations in synaptic transmission (Li et al., 2007). These genetic data suggest that although Nrxs may not be strictly required for synaptogenesis, they are crucial for the proper assembly and functional maturation of synapses. Accordingly, human genetic studies have evidenced that mutations in Nrx genes are linked to several psychiatric disorders, including autism and schizophrenia (reviewed in Südhof, 2008; Betancur et al., 2009).

There are three Nrx genes in mammals, each encoding α and β transcripts, that are further subject to alternative splicing resulting in potentially thousands of different isoforms (Ullrich et al., 1995). These molecules bind to multiple, structurally diverse postsynaptic partners. In addition to neuroligins, that were the first characterized Nrx-binding partners, Nrxs bind to a variety of other molecules, including leucine-rich repeat transmembrane proteins (LRRTMs), neurexophilins, dystroglycan, GABA_A_ receptors (GABA_A_Rs), and cerebellin-1 (Cbln1; for review, see Siddiqui and Craig, 2011). These interactions are regulated at the level of mRNA alternative splicing, generating a complex molecular “code” that may be important for synapse specification (Boucard et al., 2005; Chih et al., 2006; Uemura et al., 2010). In vertebrates, Nrxs are synthesized throughout the brain in all excitatory and inhibitory neurons (Ullrich et al., 1995). Therefore, it is generally assumed that they have a general role in synapse development and act as presynaptic hub molecules that mediate synapse maturation via selective interactions with different ligands (Südhof, 2008; Siddiqui and Craig, 2011). However, the distribution of endogenous Nrxs at distinct types of excitatory and inhibitory synapses is largely unknown, due to the lack of antibodies suitable for immunohistochemistry.

In this study, we used a pan-Nrx antiserum raised against a conserved intracellular region. Using the cerebellum as a model system, we show that Nrxs have a selective localization at distinct types of glutamatergic synapses. We also demonstrate that GABAergic interneurons regulate the synaptic expression of Nrxs during postnatal development. These differences in localization suggest that Nrxs have distinct roles in the development of glutamatergic and GABAergic synapses.

## Results

### Neurexins are present at most but not all cerebellar synapses

**Figure 1:**
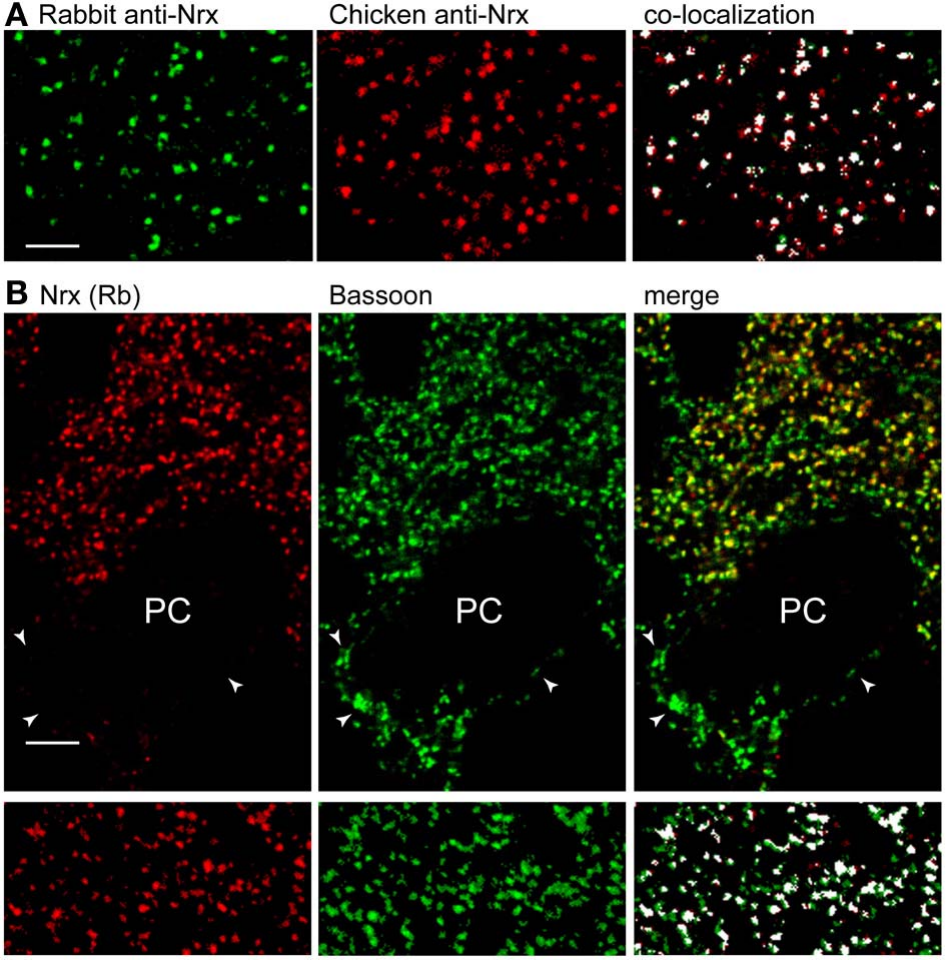
**Nrx localizes at a subset of synapses in the cerebellar cortex. (A)** Double labeling with pan-Nrx antibodies raised in rabbit (green) and chicken (red) resulted in substantial co-localization. All panels show segmented images that were processed with the Imaris co-localization algorithm (co-localized structures are shown in white). **(B)** Nrx antibodies produce punctate labeling that co-localizes extensively with the presynaptic marker bassoon. Note that bassoon-positive puncta (arrowheads) surrounding Purkinje cells (PC) are Nrx-negative. The lower panels show segmented images that were processed with the co-localization module. Scale bars: **(A)**, 3 μm; **(B)**, 5 μm.

We tested the rabbit pan-Nrx antiserum on cerebellar sections processed with different fixation techniques. We found that Nrx epitopes were sensitive to prolonged aldehyde fixation, and weak fixation protocols were therefore used to visualize Nrx immunoreactivity (see Materials and Methods). Nrx labeling was punctate, supporting synaptic localization (Figure 1). Moreover, double labeling with another pan-Nrx antibody raised in chicken (Dean et al., 2003) revealed extensive co-localization (Figure 1A), indicating that both antibodies recognize the same molecules. However, the rabbit antiserum produced a stronger labeling and was therefore used in all subsequent experiments.

To establish synaptic localization, we performed double immunofluorescence for Nrx and the presynaptic matrix protein bassoon (Figure 1B). Quantitative analysis of co-localization revealed that practically all Nrx-positive puncta in the molecular layer were associated with bassoon. However, Nrx was present in ~80% of bassoon-positive synapses (4204 co-localized puncta out of a total of 5109 bassoon-positive puncta in two different cerebella), implying that some cerebellar synapses lack Nrx. In particular, perisomatic synapses surrounding PCs were Nrx-negative, suggesting that Nrx may be absent from GABA synapses (see below).

### Neurexins are present at PF but not CF synapses

**Figure 2:**
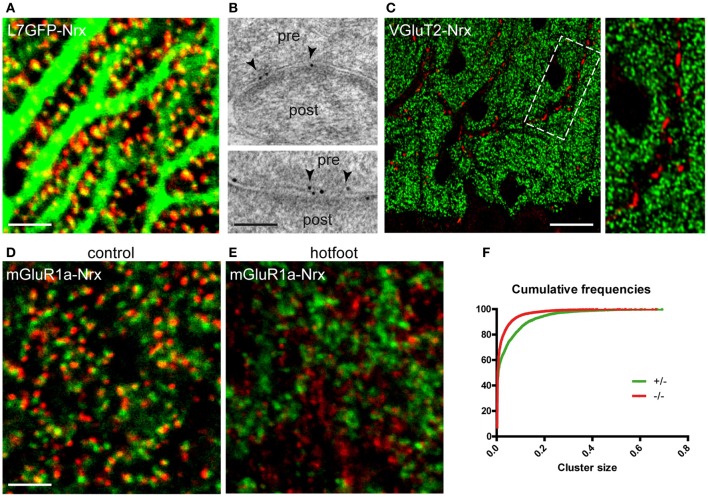
**Selective localization of Nrx at synapses made by PFs but not CFs. (A)** Nrx puncta (red) are closely apposed to Purkinje cell spines in a L7-GFP mouse cerebellum. **(B)** Immunogold labeling (arrowheads) reveals the presence of Nrx at synapses between parallel fibers (pre) and Purkinje cell spines (post). **(C)** Nrx (green) is not associated with VGluT2-positive climbing fiber terminals (red). The boxed area is shown at higher magnification in the inset. **(D,E)** Double labeling for Nrx (red) and mGluR1a (green) in the cerebellum of control **(D)** and *hotfoot* **(E)** mice. Note the altered pattern of Nrx localization in the *hotfoot* mutant. **(F)** Cumulative plot showing the reduced size of Nrx puncta in *hotfoot* mice compared to control. Scale bars: **(A,D,E)**, 3 μm; **(B)**, 100 nm; **(C)**, 18 μm.

The cerebellum provides an exquisite example of synaptic specificity. Purkinje cells (PCs), the principal neurons of the cerebellar cortex, receive glutamatergic input from two distinct sources: parallel fibers (PFs) establish synapses with spines located on the distal dendrites, whereas climbing fibers (CFs) contact spines located in the proximal dendritic domain (Yuste and Bonhoeffer, 2004; Cesa and Strata, 2009). Confocal imaging in L7-GFP mice showed that Nrx puncta were closely associated with PC spines, suggesting localization in PFs (Figure 2A). This was confirmed by immunogold labeling, showing gold particles at the presynaptic side of PF-PC synapses (Figure 2B). In contrast, we did not find Nrx signals in CF terminals, labeled with antibodies against the vesicular glutamate transporter VGluT2, indicating that in the cerebellar cortex Nrx is selectively localized at specific types of glutamatergic synapses (Figure 2C).

The presence of Nrxs at PF-PC synapses was further evidenced in double-labeling with the metabotropic glutamate receptor mGluR1a, that labels selectively PC spines (Tanaka et al., 2000). These experiments revealed that Nrx puncta were clustered opposite to mGluR1a-positive spines (Figure 2D). Notably, the pattern of Nrx expression in PF terminals was severely disrupted in mutant *hotfoot* mice, that lack the glutamate receptor GluRD2 (Mandolesi et al., 2009). In these animals, the size of Nrx puncta was significantly decreased (Kolmogorov-Smirnov, *p* < 0.001, *n* = 3) compared to the control situation (Figure 2F), as also documented by the presence of numerous small puncta (generally <0.04 μm^2^) characterized by considerably reduced brightness and fuzzy appearance (Figure 2E). These small puncta were generally not associated with PC spines (Figure 2E), suggesting that Nrx fails to form stable clusters when PFs are not connected to the appropriate postsynaptic targets. Moreover, the density of the larger Nrx puncta (0.04–0.2 μm^2^) was significantly reduced in the *hotfoot* cerebellum (mean ± SEM puncta/1000 μm^2^: control, 473.5 ± 39; *hotfoot*, 303 ± 19; *n* = 6 sampling fields from 3 mice; unpaired *t*-test, *P* = 0.0061), consistent with the robust reduction of synapses between PFs and PCs reported previously (Mandolesi et al., 2009). These data are in agreement with the idea that the integrity of PF synapses depends on a tripartite *trans*-synaptic complex comprising Nrx, the secreted glycoprotein Cbln1 and GluD2 (Matsuda et al., 2010; Uemura et al., 2010).

### Neurexins are mainly associated with developing GABA synapses

**Figure 3:**
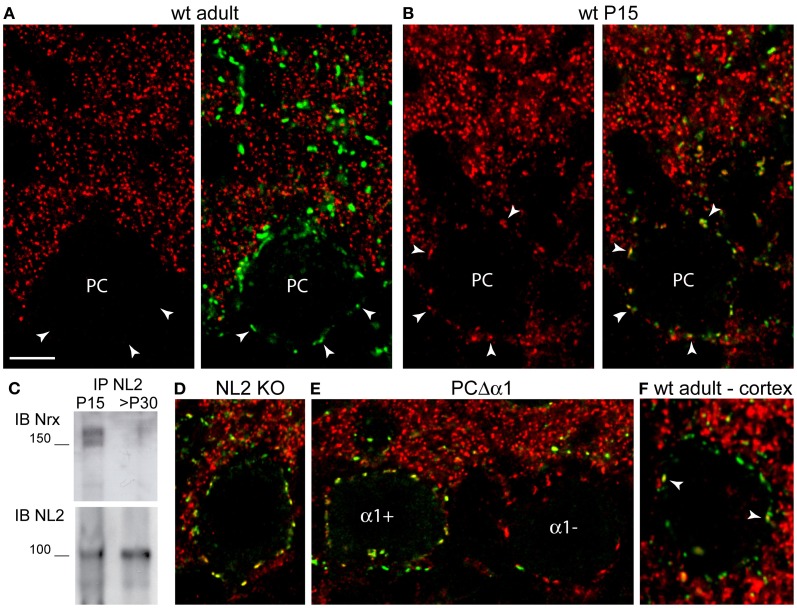
**Ontogenetic regulation of Nrxs at GABA synapses.** All confocal images show double labeling for Nrx and GABA_A_R α1 **(A,B,D,E)** or γ2 **(F)**. In all panels, labeling for Nrx is shown in red and labeling for GABA_A_Rs is shown in green. **(A)** Nrx is absent from GABA synapses in the cerebellum of an adult mouse. Arrowheads point to synaptic GABA_A_R clusters outlining the cell body of a Purkinje cell (PC). **(B)** Nrx co-distributes with GABA_A_R clusters in a P15 mouse cerebellum. **(C)** Higher amounts of Nrx are observed after co-immunoprecipitation with NL2 from synaptosomes obtained from P15 cerebella compared with >P30 cerebella. **(D)** Nrx persists at GABA synapses in a P16 NL2 KO mouse. **(E)** Nrx puncta outline the cell bodies of both GABA_A_R α1-positive and GABA_A_R α1-negative Purkinje cells in the cerebellum of a P16 PC-Δ α1 mutant mouse. **(F)** Nrx is associated with only a few (arrowheads) GABA_A_R γ2-positive, perisomatic clusters in a cortical pyramidal neuron of an adult (>P30) mouse. Scale bar: **(A,B,D–F)**, 6 μm.

We next analyzed the expression of Nrxs at GABAergic synapses. In the mature cerebellum (>P30), Nrx was below detectable levels at inhibitory synapses labeled for the GABA_A_R α1 subunit (Figure 3A) or the scaffold molecule gephyrin (not shown). Quantitative evaluation of co-distribution in the molecular layer revealed that ~3% of GABA synapses were Nrx-positive (162 co-distributed puncta out of a total of 5236 GABA_A_R-positive puncta). However, in postnatal mice (P5–P21) Nrx was found in practically all GABA synapses (co-distribution index at P15: 97%, 2810 co-distributed puncta out of a total of 2901 GABA_A_R-positive puncta), including perisomatic and axo-dendritic synapses made by basket cells and stellate cells (Figure 3B).

To validate these immunohistochemical findings, we isolated synaptosomes from cerebella of P15 and adult (>P30) mice, and we evaluated the potential of neuroligin-2 (NL2) to co-immunoprecipitate Nrx from developing and mature synapses. Because NL2 is present at GABA synapses since early stages of synaptogenesis (Patrizi et al., 2008), it can be effectively used to monitor developmental changes in the expression of interacting proteins. As expected, co-immunoprecipitation of NL2 from synaptosomes yielded considerably higher Nrx levels in juvenile than in adult mice (Figure 3C), supporting the idea that Nrx is present in developing GABA synapses, but is downregulated at the end of the synaptogenic period.

We examined whether the expression of Nrx at developing GABA synapses depends on NL2 or GABA_A_Rs, that are Nrx-binding partners at inhibitory synapses (Graf et al., 2004; Varoqueaux et al., 2004; Zhang et al., 2010). We found no defects in synaptic localization of Nrx in P16 NL2 KO mice (Figure 3D; mean ± SEM Nrx clusters/50 μm of perisomatic membrane: WT, 23.5 ± 0.45, *n* = 44 cells; KO, 24.3 ± 0.36, *n* = 57 cells; unpaired *t*-test, *p* = 0.1637). We then analyzed PC-Δα1 mice, that have a selective deletion of the GABA_A_R α1 subunit gene in PCs, resulting in complete loss of GABA_A_Rs (Briatore et al., 2010). Deletion of the α1 subunit is asynchronous among different PCs, resulting in a characteristic mosaic-like pattern, with α1-positive and α1-negative cells, in P14–P16 mice (Figure 3E). We found no differences in the synaptic localization of Nrx in α1-positive and α1-negative PCs of P16 PC-Δα1 mice (Figure 3E; mean ± SEM Nrx clusters/50 μm of somatic membrane: α1-positive PCs, 19.9 ± 0.64, *n* = 33 cells; α1-negative PCs, 19.6 ± 0.46, *n* = 32 cells; unpaired *t*-test, *p* = 0.7365). These results suggest that neither NL2 nor GABA_A_Rs are essential for Nrx localization at developing GABAergic synapses.

To establish whether the transient expression of Nrx is a general feature of GABA synapses, we analyzed co-distribution with the obligatory γ2 subunit of synaptic GABA_A_Rs in sensorimotor cortex of adult mice (Figure 3F). We found that in adult animals Nrx was associated with a small percentage of GABAergic synapses labeled with antibodies against the GABA_A_R γ2 subunit. Quantification in pyramidal neurons (*n* = 28 cells from four mice) revealed that ~30% of perisomatic synapses were Nrx-positive. Unfortunately, labeling for Nrx was generally quite challenging outside of the cerebellum, especially when combined with other antibodies, which precluded a detailed characterization of the Nrx-positive synapses. For the same reason, we could not analyze Nrx localization at developing synapses in postnatal mice. However, the limited co-distribution with GABA_A_Rs substantiates the idea that Nrxs are scarcely represented at mature GABAergic synapses.

## Discussion

The three mammalian Nrx genes undergo extensive alternative splicing in their extracellular domain, potentially generating thousands of different isoforms (Ullrich et al., 1995). Nrxs are widespread in neurons, which has led to the general assumption that Nrx isoforms could determine synaptic properties by interacting selectively with specific postsynaptic partners (Südhof, 2008; Williams et al., 2010; Siddiqui and Craig, 2011). Our present findings add a new dimension to the concept of a “molecular synaptic code” (Selimi et al., 2009), by showing that Nrxs undergo differential spatio-temporal regulation at distinct types of glutamatergic and GABAergic synapses.

We report two principal results. First, we show that Nrxs have a remarkably selective localization in PFs but not in CFs of the cerebellar cortex (Figures 2A–C). These afferents innervate distinct domains of the PC dendritic arbor, a process that depends on activity-dependent competition, as well as on differences in their molecular organization (Cesa and Strata, 2009; Kano and Hashimoto, 2009; Yuzaki, 2011). Our observations agree well with recent data showing that Nrx acts as a presynaptic ligand for the GluD2-Cbln1 complex at PF synapses (Matsuda et al., 2010; Uemura et al., 2010). Extensive evidence indicates that Cbln1, which is secreted by PFs, acts as a bi-directional receptor for both GluD2 and Nrx, thus providing a physical linkage between the postsynaptic density in PC spines and the active zone in PF terminals (Uemura et al., 2010; Yuzaki, 2011). Consistent with this idea, we found that Nrx clusters were altered in the cerebellum of *hotfoot* mice (Figure 2E), that lack GluD2. First, there was a significant reduction in the density of the larger Nrx puncta, that presumably corresponded to synapses between PFs and PCs. Second, many small clusters unrelated to PC spines became apparent in the molecular layer, suggesting that the presynaptic localization of Nrx depends on the structural integrity of PF synapses and proper molecular interactions mediated by the GluD2-Cbln1 complex. On a more general level, the selective localization of Nrx at PF but not CF synapses indicates that these glutamatergic contacts depend on *trans*-synaptic interactions mediated by distinct complexes of adhesion molecules. While the Nrx/Cbln1/GluD2 triad likely represents a “molecular code” for PF synapses (Uemura et al., 2010), the identity of adhesion molecules expressed at CF synapses remains to be determined. Synaptic protein profiling (Selimi et al., 2009) and other proteomic strategies may help to decipher signaling pathways that are selectively involved in the specification of these glutamatergic contacts.

Another important finding was that Nrxs undergo a developmentally regulated expression at GABA synapses. The situation in the cerebellar cortex is paradigmatic. Here Nrx was associated with GABA synapses during the entire period of postnatal development, but was strongly downregulated at the end of synaptogenesis, resulting in undetectable levels in mature circuits (Figures 3A,B). This transient expression is in perfect agreement with *in vitro* analyses, showing that overexpression of Nrxs in cultured neurons did not impair existing GABA synapses, but affected selectively the properties of developing contacts (Zhang et al., 2010). Together, these data suggest that Nrx may act to regulate the functional maturation of developing GABAergic synapses. Interestingly, a recent study in *Caenorhabditis* showed that Nrx and NL mediate a retrograde synaptic signal that inhibits neurotransmitter release by adjusting the rate and duration of synaptic vesicle release (Hu et al., 2012). Studies on cultured neurons also have demonstrated that Nrxs suppress GABAergic synaptic transmission by direct binding to GABA_A_Rs (Zhang et al., 2010). Therefore, we speculate that the ontogenetically regulated expression of Nrx at GABA synapses may represent a mechanism to adjust the levels of transmission at a time when GABA has a strong influence over the developmental assembly of neuronal circuits (Akerman and Cline, 2007; Huang and Scheiffele, 2008; Wang and Kriegstein, 2009).

The expression profile in neocortex was quite different, as Nrx was retained in a subset of GABA synapses in adult mice (Figure 3F). It is of note that Nrxs bind directly to α1-GABA_A_Rs (Zhang et al., 2010), that are the unique type of GABA_A_R in PCs and have a more limited distribution in neocortical neurons. It is possible that Nrxs may be differently regulated at distinct types of inhibitory synapses characterized by different GABA_A_R subtypes. Unfortunately, we have been unable to address this question due to the difficulties in detecting endogenous Nrxs. It should also be mentioned that the pan-Nrx antiserum used in this study was raised against a C-terminal amino acid sequence of Nrx1 that is largely conserved in the Nrx2 and Nrx3 isoforms (see Materials and Methods). While immunoblot analyses have shown that the antiserum recognizes all Nrx isoforms (Bottos et al., 2009), we cannot exclude that our observations are biased by higher affinity of the antiserum for Nrx1 than for the other Nrxs. The situation is also complicated by the fact that other than the original *in situ* hybridization mapping, that revealed differential but overlapping distribution of Nrx isoforms in distinct classes of neurons (Ullrich et al., 1995), very little has become available about the expression of the different isoforms and their regulation at the cellular and synaptic levels.

Our current results suggest that the localization of Nrx at developing GABA synapses is not influenced by binding to its postsynaptic ligands NL2 and GABA_A_Rs (Figures 3D,E). However, our observations in NL2 KO mice may be confounded by compensatory changes involving other NL isoforms. Indeed, we have observed a strong upregulation of NL3 and NL4 in PCs of NL2 KO mice (data not shown). Moreover, cellular imaging after artificial expression of tagged Nrx isoforms in cortical interneurons demonstrated that the presynaptic localization of Nrx1β depends on binding to postsynaptic ligands (Fu and Huang, 2010). On the other hand, no GABA_A_Rs are present in PCs of PC-Δ α1 mice (Briatore et al., 2010), suggesting that binding to GABA_A_Rs is not essential for presynaptic stabilization of Nrxs.

While there is no doubt that endogeneous Nrxs are presynaptic, it has been proposed that these adhesion molecules may partly localize postsynaptically (Taniguchi et al., 2007). Unfortunately, postembedding electron microscopy yielded relatively weak labeling, precluding quantitative analysis of gold particle distribution at PF-PC synapses. However, the precise apposition of NRX-positive puncta with PC spines evidenced by confocal microscopy (Figure 2D) is consistent with a predominantly presynaptic localization.

In conclusion, our findings suggest that synapses are specified by molecular interactions that involve both Nrx-dependent and Nrx-independent mechanisms. Moreover, the complex spatio-temporal expression profile reported here suggests that ontogenetic regulation of Nrxs is of crucial importance for determining how synapses form and acquire their functional maturation.

## Materials and methods

### Mice

Most of the experiments described in this study were performed on WT mice of the C57BL/6 strain and transgenic mice expressing GFP selectively in PCs (L7-GFP mice). In addition, three lines of mutant mice were used. *Hotfoot* mice are spontaneous mutants with a deletion of the glutamate receptor GluRD2 (Lalouette et al., 1998). GluD2 is expressed selectively in PC spines innervated by PFs and is crucial for the formation and maintenance of PF synapses (Kashiwabuchi et al., 1995; Yuzaki, 2003). Like GluRD2 knockout (KO) mice, *hotfoot* mutants have a reduced number of synapses between PFs and PCs (Mandolesi et al., 2009). The generation of PC-Δ α1 mice has been described elsewhere (Briatore et al., 2010). Deletion of the GABA_A_R α1 subunit gene was driven by Cre recombinase expressed under control of the L7 promoter, which resulted in a characteristic mosaic pattern, with α1-positive and α1-negative cells, in P14–P16 mice (Figure 3E). Finally, NL2 KO mice (Varoqueaux et al., 2006) were kindly provided by Nils Brose (Max-Planck-Institute of Experimental Medicine and Center for Molecular Physiology of the Brain, Göttingen, Germany).

The experimental procedures were designed in accordance with national (Legislative Decree 116/92 and law n. 413/1993) and international (Directive 86/609/EEC and the recommendation 2007/526/EC from the European Community) laws and policies, and approved by the Italian Ministry of Health (Department of Public Veterinary Health) and by the ethical committee of Turin University.

### Antibodies

We used an affinity-purified, pan-Nrx antiserum produced in rabbits as described in detail elsewhere (Bottos et al., 2009). This antibody was raised against the peptide AKSANKNKKNKDKEYYV located in the intracellular region of the α and β isoforms of mouse Nrx1. This region has high homology to Nrx2 (PKTPSKAKKNKDKEYYV) and Nrx3 (SKSGHKKQKNKDKEYYV). Consistent with this, the antiserum recognized all Nrx isoforms in immunoblotting of transfected HeLa cells. Another antiserum (kindly provided by Peter Scheiffele, Biozentrum, Basel, Switzerland) was raised in chicken against a recombinant neurexin-GST fusion protein containing the cytoplasmic tail of Nrx1 (Dean et al., 2003). This antiserum recognized two bands with apparent molecular weights corresponding to α and β Nrxs in western blots of total cerebellar lysates. A rabbit antiserum against NL2 was obtained from Synaptic Systems (cat. no. 129203). Guinea pig antisera against the vesicular glutamate transporter VGluT2 (Chemicon, AB2251) and the metabotropic glutamate receptor mGluR1a (kindly provided by Masahiko Watanabe, Hokkaido University, Sapporo, Japan) were used to label respectively CF terminals and PC spines (Tanaka et al., 2000; Fremeau et al., 2001). GABA synapses were identified with guinea pig antibodies against the α1 and γ2 subunits of GABA_A_Rs (kindly provided by Jean-Marc Fritschy, University of Zurich, Switzerland). The monoclonal antibody against bassoon was obtained from Stressgen (cat. no. VAM-PS003).

### Immunofluorescence and confocal microscopy

Detection of Nrx required a brief fixation protocol described in detail elsewhere (protocol B in Schneider Gasser et al., 2006; see also Patrizi et al., 2008). In some cases, mice were perfused with 2% formaldehyde, and the brains were postfixed in the same fixative overnight. This fixation protocol allowed simultaneous detection of Nrx and VGluT2. Confocal images were acquired with a laser scanning confocal microscope (Zeiss LSM5 Pascal) using the multitrack mode to avoid fluorescence crosstalk. Synaptic puncta were analyzed on images acquired with a ×100 oil-immersion objective (1.4 NA) at a magnification of 8.1 × 10^−3^ μm^2^/pixel, and the pinhole set at 1 Airy unit. The images were processed with the image-analysis program Imaris (release 4.2; Bitplane). Clusters were quantified on segmented images with NIH Image J software. Co-localization was estimated on segmented images with the Imaris co-localization module (see Viltono et al., 2008). The density of perisomatic clusters was determined by counting manually Nrx puncta surrounding the cell body of PCs.

### Immunogold labeling

Juvenile (P17) mice (*n* = 2) were perfused with 2% formaldehyde and 0.1% glutaraldehyde in sodium acetate buffer (pH 6) for 2 min, followed by 1 h perfusion with 2% formaldehyde and 0.1% glutaraldehyde in 0.1 M borate buffer (pH 9). Brains were postfixed in the second fixative solution overnight. Tissue blocks from the cerebellar vermis were freeze-substituted and embedded in Lowicryl HM20. Ultrathin sections were processed for the immunogold method using as secondary antibodies goat Fab fragments coupled to 10 nm colloidal gold particles (Sassoè-Pognetto and Ottersen, 2000).

### Synaptosomal preparation and co-immunoprecipitation

Synaptosome extraction was performed using the Syn-PER Reagent (Thermo Scientific) following the manufacturer's protocol. Frozen cerebella obtained from P15 and adult (>P30) mice were disgregated with a tissue potter and lysed on ice with Syn-PER Reagent (10 ml per gram of tissue) added with protease and phosphatase inhibitors (50 μg/mL pepstatin, 50 μg/mL leupeptin, 10 μg/mL aprotinin, 1 mM PMSF, 100 μM ZnCl_2_, 1 mM Na orthovanadate, and 10 mM NaF). The solution was centrifuged at 1200 g for 10 min at 4°C, the pellet was discarded and the remaining supernatant was centrifuged at 10,000 g for 45 min at 4°C. The resulting pellet was resuspended in 2 ml of lysis buffer [10 mM Tris HCl, pH 7.5; 150 mM NaCl; 5 mM EDTA, pH 8; 10% glycerol; 1% Triton X-100; and 1% 3-[(3-Cholamidopropyl)dimethylammonio]1-propanesulfonate (CHAPS)] with protease and phosphatase inhibitors (50 μg/mL pepstatin, 50 μg/mL leupeptin, 10 μg/mL aprotinin, 1 mM PMSF, 100 μM ZnCl_2_, 1 mM Na orthovanadate, and 10 mM NaF). After quantification with the BCA Protein Assay Reagent Kit (Pierce Chemical Co.), samples (5 mg of total proteins) were precleared with protein A-Sepharose (Amersham Biosciences) and incubated 1 h with the rabbit anti-Nrx antibody (1.5 μg/mg) or the rabbit anti-NL2 antibody (2.5 μg/mg). Immune complexes were recovered on protein A-Sepharose overnight and washed 4 times. Proteins were separated by 4–15% SDS/PAGE electrophoresis gel, transferred to polyvinylidene difluoride membrane (Millipore), and detected by immunoblot. Immunoreactive proteins were identified with an HRP-conjugated secondary antibody (Jackson ImmunoResearch) and visualized by an ECL system (Amersham Biosciences).

### Conflict of interest statement

The authors declare that the research was conducted in the absence of any commercial or financial relationships that could be construed as a potential conflict of interest.

## References

[B1] Akerman C. J. Cline H. T. (2007). Refining the roles of GABAergic signaling during neural circuit formation. Trends Neurosci. 30, 382–389 10.1016/j.tins.2007.06.002 17590449

[B2] Betancur C. Sakurai T. Buxbaum J. D. (2009). The emerging role of synaptic cell-adhesion pathways in the pathogenesis of autism spectrum disorders. Trends Neurosci. 32, 402–412 10.1016/j.tins.2009.04.003 19541375 PMC10354373

[B3] Bottos A. Destro E. Rissone A. Graziano S. Cordara G. Assenzio B. (2009). The synaptic proteins neurexins and neuroligins are widely expressed in the vascular system and contribute to its functions. Proc. Natl. Acad. Sci. U.S.A. 106, 20782–20787 10.1073/pnas.0809510106 19926856 PMC2791601

[B4] Boucard A. A. Chubykin A. A. Comoletti D. Taylor P. Südhof T. C. (2005). A splice code for trans-synaptic cell adhesion mediated by binding of neuroligin 1 to alpha- and beta-neurexins. Neuron 48, 229–236 10.1016/j.neuron.2005.08.026 16242404

[B5] Briatore F. Patrizi A. Viltono L. Sassoè-Pognetto M. Wulff P. (2010). Quantitative organization of GABAergic synapses in the molecular layer of the mouse cerebellar cortex. PLoS ONE 5:e12119 10.1371/journal.pone.0012119 20711348 PMC2920831

[B6] Cesa R. Strata P. (2009). Axonal competition in the synaptic wiring of the cerebellar cortex during development and in the mature cerebellum. Neuroscience 162, 624–632 10.1016/j.neuroscience.2009.02.061 19272433

[B7] Chih B. Gollan L. Scheiffele P. (2006). Alternative splicing controls selective trans-synaptic interactions of the neuroligin-neurexin complex. Neuron 51, 171–178 10.1016/j.neuron.2006.06.005 16846852

[B8] Craig A. M. Kang Y. (2007). Neurexin-neuroligin signaling in synapse development. Curr. Opin. Neurobiol. 17, 43–52 10.1016/j.conb.2007.01.011 17275284 PMC2820508

[B9] Dean C. Scholl F. G. Choih J. DeMaria S. Berger J. Isacoff E. (2003). Neurexin mediates the assembly of presynaptic terminals. Nat. Neurosci. 6, 708–716 10.1038/nn1074 12796785 PMC1646425

[B10] Fremeau R. T. Jr. Troyer M. D. Pahner I. Nygaard G. O. Tran C. H. Reimer R. J. (2001). The expression of vesicular glutamate transporters defines two classes of excitatory synapse. Neuron 31, 247–260 10.1016/S0896-6273(01)00344-0 11502256

[B11] Fu Y. Huang Z. J. (2010). Differential dynamics and activity-dependent regulation of alpha- and beta-neurexins at developing GABAergic synapses. Proc. Natl. Acad. Sci. U.S.A. 107, 22699–22704 10.1073/pnas.1011233108 21149722 PMC3012487

[B12] Graf E. R. Zhang X. Jin S. X. Linhoff M. W. Craig A. M. (2004). Neurexins induce differentiation of GABA and glutamate postsynaptic specializations via neuroligins. Cell 119, 1013–1026 10.1016/j.cell.2004.11.035 15620359 PMC2826211

[B13] Hu Z. Hom S. Kudze T. Tong X. J. Choi S. Aramuni G. (2012). Neurexin and neuroligin mediate retrograde synaptic inhibition in C. elegans. Science 337, 980–984 10.1126/science.1224896 22859820 PMC3791080

[B14] Huang Z. J. Scheiffele P. (2008). GABA and neuroligin signaling: linking synaptic activity and adhesion in inhibitory synapse development. Curr. Opin. Neurobiol. 18, 77–83 10.1016/j.conb.2008.05.008 18513949 PMC3988756

[B15] Ichtchenko K. Hata Y. Nguyen T. Ullrich B. Missler M. Moomaw C. (1995). Neuroligin 1: a splice site-specific ligand for beta-neurexins. Cell 81, 435–443 10.1016/0092-8674(95)90396-8 7736595

[B16] Kano M. Hashimoto K. (2009). Synapse elimination in the central nervous system. Curr. Opin. Neurobiol. 19, 154–161 10.1016/j.conb.2009.05.002 19481442

[B17] Kashiwabuchi N. Ikeda K. Araki K. Hirano T. Shibuki K. Takayama C. (1995). Impairment of motor coordination, Purkinje cell synapse formation, cerebellar long-term depression in GluR delta 2 mutant mice. Cell 81, 245–252 10.1016/0092-8674(95)90334-8 7736576

[B18] Lalouette A. Guénet J. L. Vriz S. (1998). Hotfoot mouse mutations affect the delta 2 glutamate receptor gene and are allelic to lurcher. Genomics 50, 9–13 10.1006/geno.1998.5314 9628817

[B19] Li J. Ashley J. Budnik V. Bhat M. A. (2007). Crucial role of Drosophila neurexin in proper active zone apposition to postsynaptic densities, synaptic growth, and synaptic transmission. Neuron 55, 741–755 10.1016/j.neuron.2007.08.002 17785181 PMC2039911

[B20] Mandolesi G. Autuori E. Cesa R. Premoselli F. Cesare P. Strata P. (2009). GluRdelta2 expression in the mature cerebellum of hotfoot mice promotes parallel fiber synaptogenesis and axonal competition. PLoS ONE 4:e5243 10.1371/journal.pone.0005243 19370152 PMC2666267

[B21] Matsuda K. Miura E. Miyazaki T. Kakegawa W. Emi K. Narumi S. (2010). Cbln1 is a ligand for an orphan glutamate receptor delta2, a bidirectional synapse organizer. Science 328, 363–368 10.1126/science.1185152 20395510

[B22] Missler M. Zhang W. Rohlmann A. Kattenstroth G. Hammer R. E. Gottmann K. (2003). Alpha-neurexins couple Ca2+ channels to synaptic vesicle exocytosis. Nature 423, 939–948 10.1038/nature01755 12827191

[B23] Nam C. I. Chen L. (2005). Postsynaptic assembly induced by neurexin-neuroligin interaction and neurotransmitter. Proc. Natl. Acad. Sci. U.S.A. 102, 6137–6142 10.1073/pnas.0502038102 15837930 PMC1087954

[B24] Patrizi A. Scelfo B. Viltono L. Briatore F. Fukaya M. Watanabe M. (2008). Synapse formation and clustering of neuroligin-2 in the absence of GABAA receptors. Proc. Natl. Acad. Sci. U.S.A. 105, 13151–13156 10.1073/pnas.0802390105 18723687 PMC2529038

[B25] Sassoè-Pognetto M. Ottersen O. P. (2000). Organization of ionotropic glutamate receptors at dendrodendritic synapses in the rat olfactory bulb. J. Neurosci. 20, 2192–2201 10704494 10.1523/JNEUROSCI.20-06-02192.2000 PMC6772496

[B26] Schneider Gasser E. M. Straub C. J. Panzanelli P. Weinmann O. Sassoè-Pognetto M. Fritschy J. M. (2006). Immunofluorescence in brain sections: simultaneous detection of presynaptic and postsynaptic proteins in identified neurons. Nat. Protoc. 1, 1887–1897 10.1038/nprot.2006.265 17487173

[B27] Selimi F. Cristea I. M. Heller E. Chait B. T. Heintz N. (2009). Proteomic studies of a single CNS synapse type: the parallel fiber/purkinje cell synapse. PLoS Biol. 7:e83 10.1371/journal.pbio.1000083 19402746 PMC2672601

[B28] Shen K. Scheiffele P. (2010). Genetics and cell biology of building specific synaptic connectivity. Annu. Rev. Neurosci. 33, 473–507 10.1146/annurev.neuro.051508.135302 20367446 PMC3082953

[B29] Siddiqui T. J. Craig A. M. (2011). Synaptic organizing complexes. Curr. Opin. Neurobiol. 21, 132–143 10.1016/j.conb.2010.08.016 20832286 PMC3016466

[B30] Südhof T. C. (2008). Neuroligins and neurexins link synaptic function to cognitive disease. Nature 455, 903–911 10.1038/nature07456 18923512 PMC2673233

[B31] Tanaka J. Nakagawa S. Kushiya E. Yamasaki M. Fukaya M. Iwanaga T. (2000). Gq protein alpha subunits Galphaq and Galpha11 are localized at postsynaptic extra-junctional membrane of cerebellar Purkinje cells and hippocampal pyramidal cells. Eur. J. Neurosci. 12, 781–792 10.1046/j.1460-9568.2000.00959.x 10762307

[B32] Taniguchi H. Gollan L. Scholl F. G. Mahadomrongkul V. Dobler E. Limthong N. (2007). Silencing of neuroligin function by postsynaptic neurexins. J. Neurosci. 27, 2815–2824 10.1523/JNEUROSCI.0032-07.2007 17360903 PMC2839889

[B33] Uemura T. Lee S. J. Yasumura M. Takeuchi T. Yoshida T. Ra M. (2010). Trans-synaptic interaction of GluRdelta2 and Neurexin through Cbln1 mediates synapse formation in the cerebellum. Cell 141, 1068–1079 10.1016/j.cell.2010.04.035 20537373

[B34] Ullrich B. Ushkaryov Y. A. Südhof T. C. (1995). Cartography of neurexins: more than 1000 isoforms generated by alternative splicing and expressed in distinct subsets of neurons. Neuron 14, 497–507 10.1016/0896-6273(95)90306-2 7695896

[B35] Varoqueaux F. Aramuni G. Rawson R. L. Mohrmann R. Missler M. Gottmann K. (2006). Neuroligins determine synapse maturation and function. Neuron 51, 741–754 10.1016/j.neuron.2006.09.003 16982420

[B36] Varoqueaux F. Jamain S. Brose N. (2004). Neuroligin 2 is exclusively localized to inhibitory synapses. Eur. J. Cell Biol. 83, 449–456 10.1078/0171-9335-00410 15540461

[B37] Viltono L. Patrizi A. Fritschy J. M. Sassoè-Pognetto M. (2008). Synaptogenesis in the cerebellar cortex: differential regulation of gephyrin and GABAA receptors at somatic and dendritic synapses of Purkinje cells. J. Comp. Neurol. 508, 579–591 10.1002/cne.21713 18366064

[B38] Waites C. L. Craig A. M. Garner C. C. (2005). Mechanisms of vertebrate synaptogenesis. Annu. Rev. Neurosci. 28, 251–274 10.1146/annurev.neuro.27.070203.144336 16022596

[B38a] Wang D. D. Kriegstein A. R. (2009). Defining the role of GABA in cortical development. J. Physiol. 587, 1873–1879 10.1113/jphysiol.2008.167635 19153158 PMC2689328

[B39] Williams M. E. de Wit J. Ghosh A. (2010). Molecular mechanisms of synaptic specificity in developing neural circuits. Neuron 68, 9–18 10.1016/j.neuron.2010.09.007 20920787 PMC3327884

[B40] Yuste R. Bonhoeffer T. (2004). Genesis of dendritic spines: insights from ultrastructural and imaging studies. Nat. Rev. Neurosci. 5, 24–34 10.1038/nrn1300 14708001

[B41] Yuzaki M. (2003). The delta2 glutamate receptor: 10 years later. Neurosci. Res. 46, 11–22 10.1016/S0168-0102(03)00036-1 12725908

[B42] Yuzaki M. (2011). Cbln1 and its family proteins in synapse formation and maintenance. Curr. Opin. Neurobiol. 21, 215–220 10.1016/j.conb.2011.01.010 21342763

[B43] Zhang C. Atasoy D. Araç D. Yang X. Fucillo M. V. Robison A. J. (2010). Neurexins physically and functionally interact with GABA(A) receptors. Neuron 66, 403–416 10.1016/j.neuron.2010.04.008 20471353 PMC3243752

[B44] Zhang W. Rohlmann A. Sargsyan V. Aramuni G. Hammer R. E. Südhof T. C. (2005). Extracellular domains of alpha-neurexins participate in regulating synaptic transmission by selectively affecting N- and P/Q-type Ca2+ channels. J. Neurosci. 25, 4330–4342 10.1523/JNEUROSCI.0497-05.2005 15858059 PMC6725120

